# Gene-environment interaction between body mass index and *transforming growth factor beta 1 *(*TGFβ1*) gene in knee and hip osteoarthritis

**DOI:** 10.1186/ar4214

**Published:** 2013-04-18

**Authors:** Stella G Muthuri, Sally Doherty, Weiya Zhang, Rose A Maciewicz, Kenneth R Muir, Michael Doherty

**Affiliations:** 1 Division of Epidemiology and Public Health, University of Nottingham, Clinical Sciences Building, Nottingham City Hospital, Nottingham, NG5 1PB, UK; 2 Academic Rheumatology, University of Nottingham, Clinical Sciences Building, Nottingham City Hospital, Nottingham, NG5 1PB, UK; 3 Respiratory & Inflammation, AstraZeneca, Charnwood R&D, Loughborough, Leicestershire, LE11 5RH, UK; 4 Heath Services Research Institute, Warwick Medical School and Warwick University, Coventry CV4 7AL, UK

## Abstract

**Introduction:**

The objective was to investigate potential gene-environment interaction between body mass index (BMI) and each of eight *TGFβ1 *polymorphisms in knee and hip osteoarthritis (OA).

**Methods:**

We conducted a case-control study of Caucasian men and women aged 45 to 86 years from Nottingham, United Kingdom (Genetics of OA and Lifestyle (GOAL) study). Cases had clinically severe symptoms and radiographic knee or hip OA; controls had no symptoms and no radiographic knee/hip OA. We used logistic regression to investigate the association of *TGFβ1 *polymorphisms and OA when stratifying by BMI. Knee and hip OA were analyzed separately with adjustment for potential confounders. Additive and multiplicative interactions were examined.

**Results:**

2,048 cases (1,042 knee OA, 1,006 hip OA) and 967 controls were studied. For hip OA, the highest risk was in overweight (BMI ≥25 kg/m^2^) individuals with the variant allele of single-nucleotide polymorphism (SNP) rs1800468 (odds ratio (OR) 2.21, 95% confidence interval (CI) 1.55, 3.15). Evaluation of gene-environment interaction indicated significant synergetic interaction (relative excess risk due to interaction (RERI) = 0.93, synergy index (SI) = 4.33) with an attributable proportion due to interaction (AP) of 42% (AP = 0.42; 95% CI 0.16, 0.68). Multiplicative interaction was also significant (OR for interaction (ORINT) = 2.27, *P *= 0.015). For knee OA, the highest risk was in overweight individuals with homozygous genotype 11 of SNP rs2278422 (OR = 6.95, *P *<0.001). In contrast, the variant allele indicated slightly lower risks (OR = 4.72, *P *<0.001), a significant antagonistic interaction (RERI = -2.66, SI = 0.59), AP = -0.56 (95%CI -0.94, -0.17) and a significant multiplicative interaction (ORINT = 0.47, *P *= 0.013).

**Conclusion:**

*TGFβ1 *gene polymorphisms interact with being overweight to influence the risk of large joint OA.

## Introduction

Osteoarthritis (OA) is a complex disorder with genetic and environmental risk factors both contributing to its development and progression [1]. Obesity is one of the strongest environmental risk factors for knee OA [2] and is considered to be moderately associated with hip OA [3]. Several chromosomal loci and gene variations have also been reported to influence OA disease processes [4,5]. *Transforming growth factor beta 1 *(*TGFβ1*) gene is one potential candidate gene for OA. *TGFβ1 *is considered essential for cartilage integrity and is found at high levels in normal cartilage but has reduced expression in cartilage in OA [6]. Furthermore, increased endogenous *TGFβ1 *expression is reported during osteophyte formation [6,7], which reportedly stabilise joints in OA and reduce structural progression [8].

Gene association studies have reported independent associations with peripheral OA and spinal osteophytosis. Within the *TGFβ1 *gene, single nucleotide polymorphisms (SNPs), rs2278422 and rs8179181, have been found to have a possible role in susceptibility to knee and hip OA in a British Caucasian population [9], and a variation on position 29 (Leu10Pro) or SNP rs1982073 has been implicated with spinal osteophytosis in Japanese postmenopausal women [10]. Furthermore, increased *TGFβ1 *expression has been associated with body mass index (BMI) and abdominal adipose tissue in morbid obesity [11]. The mechanism by which body weight may exert its effect on *TGFβ1 *is unclear. Evidence from *in vitro *experiments with human and animal culture cells as well as *in vivo *animal studies have shown an increased expression of *TGFβ1 *in response to mechanical stimuli in a number of cell and tissue types [12,13], implying that excessive biomechanical loading of joints may stimulate the up-regulation of endogenous *TGFβ1 *and thus, predispose to the development or progression of OA.

Environmental and genetic factors may both play a role in large joint OA but no epidemiological studies have described possible gene environmental interactions. Evidence from twin studies estimate that genetic factors influence 39% to 65% of hand and knee OA in women and about 60% of hip OA [5]. This implies that OA is not only under the influence of genetic factors but also environmental factors and that these may modify the penetrance of OA susceptibility genes, even those showing Mendelian gene inheritance. Cartilage loss and osteophytes are central features in OA and weight-bearing may impact this, therefore, we asked whether there were any interactions in polymorphisms in genes involved in joint tissue integrity (for example, *TGFβ1*) and environmental factors influencing weight-bearing (for example, weight). We utilised data from the Genetics of OA and Lifestyle (GOAL) cohort, where both body weight [14] and polymorphisms in *TGFβ1 *[9] have been shown to be independent risk factors, to investigate whether being overweight interacts with *TGFβ1 *gene polymorphisms to influence the risk of hip or knee OA.

## Materials and methods

### Participants

The GOAL study was designed as a case-control study to characterise risk factors in large joint OA. The study was approved by the Nottingham Research Ethics Committee and fully informed consent was obtained. Participants were unrelated Caucasian men and women, aged between 45 and 86 years and resident in Nottinghamshire. The recruitment of cases and controls took place between 2002 and 2006 and details of the study are published elsewhere [9,15,16]. In brief, cases were selected if they had clinically significant symptomatic OA of the hip or knee, sufficient to warrant hospital referral. They were recruited if they had either undergone joint replacement, were on the orthopaedic waiting list or had been referred with symptomatic knee OA to the Nottingham knee OA clinic. Controls were recruited from hospital intravenous urography (IVU) waiting lists and frequency-matched to cases by age (± 2 years) and gender. Controls with no evidence of hip OA on review of their IVU radiographs were invited to take part in the study. Only individuals that met the study inclusion criteria were sampled [9,15,16]. The participation rate of eligible cases and controls was 62% and 56%, respectively. This included 1,042 knee OA cases, 1,006 hip OA cases and 1,123 non OA controls.

### Data collection

The GOAL study collected data using an interview-administered questionnaire and clinical examination. The questionnaire collected information on socio-demographic factors, employment history, occupational activity, and significant injury, and also contained detailed questions on other risk factors for OA. Weight (kg) and height (cm) were measured by a trained research nurse during the clinical examination. New knee, hand and pelvis radiographs were taken at the clinical examination unless the participant had undergone radiography not more than two years prior to the study or had undergone total joint replacement (TJR). Radiographic assessment and grading for features of OA have been described in detail elsewhere [9,15,16].

### Genotyping

Genomic DNA was extracted from whole blood using Gentra PureGene kit (Qiagen). TaqMan^® ^allelic discrimination genotyping method was applied to detect genotype polymorphisms at the *TGFβ1 *locus as described previously [9].

### Exposure variables

Body mass index (BMI) was calculated in kg/m^2 ^[14]. Possible confounding factors included age, sex, bone mineral density (BMD) , nodal OA, significant joint injury and occupational risk factors. Calcaneal bone density (single heel determined by hand dominance) in g/cm^2 ^was measured using DXA (Norland Apollo 501A00Z) and the age-adjusted z-score was used for BMD in the analysis. The presence of interphalangeal nodes was determined during the clinical examination and nodal OA was defined as Heberden's and/or Bouchard's nodes present in at least two x-rays of each hand [16]. Participants self-reported previous significant joint injury to the knee and hip joint. Also included in the interviewer-administered questionnaire were detailed questions about jobs held since leaving school. For each job reported, information was sought on tasks performed on an average working day that involved 12 specified occupational activities such as kneeling and squatting as well as the weekly frequency of lifting different levels of weights. For this analysis, the longest held occupation was used. We truncated occupational exposure for patients who had undergone TJR so that the longest held job prior to TJR was selected. This was also truncated in all controls to the longest job held three years earlier before the recruitment date [14]. Established occupational risk factors for knee and hip OA including kneeling, squatting, carrying out heavy work whilst standing (≥1 hour), lifting 25 kg (10+ times per week), lifting 50 kg or 100 kg (1+ times per week), were then scored for each individual. Subsequently, each score was summed (maximum score 6) to estimate occupational risks.

### Statistical analysis

Analyses were conducted separately on knee and hip OA using SPSS version 18. For the present analysis, all knee OA (n = 1,042) and hip OA (n = 1,006) patients, and controls (n = 967) who had no symptoms and no radiographic evidence of hip or knee OA (defined as Kellgren-Lawrence score (KL) ≤2 at the knee and KL ≤3 at the hip joint) were included.

Participants were stratified into four strata according to genotype (G) and BMI (E): G-/E-, G+/E-, G-/E+ and G+/E+. Genotype 11 was defined as negative (coded as 0) and genotype 12 or 22 was defined as positive (coded as 1). BMI <25 kg/m^2 ^was defined as negative (coded as 0) and BMI ≥25 kg/m^2 ^was defined as positive (coded as 1). The ddds ratio (OR) and 95% CI was calculated for G+/E-, G-/E+ and G+/E+ compared with G-/E-. The study was powered to detect an OR for interaction (ORINT) of 5 based on the multiplicative model, that is, OR_11 _> OR_01 _× OR_10_. Unconditional logistic regression was used to adjust for confounding. Two models were developed: one to only adjust for age (continuous variable) and gender (model 1); the other to adjust for all putative risk factors including age, gender, BMD (continuous variable), significant joint injury (yes/no), nodal OA (yes/no) and occupational risk factors (no risk factors, and 1, 2 and 3 or more knee occupational risk factors). All statistical tests were two-sided (Wald's c² statistic), with 5% significance level.

To examine interaction, between gene polymorphisms and BMI, multiplicative and additive models of interaction were used. To test for multiplicative interactions, ORs were first examined across different strata and then an interaction term (genotype*BMI) was included in a logistic regression model. Multivariate models were used to adjust for covariates listed in association analyses and a *P-*value below 0.05 (likelihood ratio test, LRT) was used to indicate a significant interaction. In addition, we assessed departure from additivity of absolute effects as recommended by Rothman [17]. Andersson [18] has described a detailed calculation method of additive interaction, an SAS programme, including instructions for how to adjust the programme for use in SPSS, and an Excel calculator available from EpiNET [19] which we used to estimate the three indicators of interaction and 95% CI. The method follows the approach proposed by Lundberg *et al*. [20] to calculate three measures of additive interactions: relative excess risk due to interaction (RERI), attributable proportion (AP) and synergy index (SI), and a method suggested by Hosmer and Lemeshow [21] to estimate 95% CI. We considered RERI and AP to be equal to 0 and SI equal to 1 to indicate absence of additive interactions [18]. Conversely, additive interaction is considered present if RERI and AP do not equal 0 and SI exceeds unity. Furthermore, if RERI is greater than 0, this denotes a synergetic interaction, which implies that the combined action between two exposures in an additive model is greater than the sum of the individual effects. However, if RERI is less than 0, it implies an antagonistic interaction - meaning that in the presence of two exposures in an additive model, the action of one exposure variable diminishes the effect of the other [17].

## Results

Table 1 shows the characteristics of the OA cases and controls. Overall, OA cases compared to controls were significantly older, had a higher BMD, and were more likely to be obese, to have nodal OA, to report previous injury and to have exposure to occupational risk factors.

**Table 1:** Characteristics of the study population

	Controls	Knee OA cases	Hip OA cases
Number	967	1042	1006
Age, years (SD)	63.5 (8.5)	68.1 (7.4)*	67.6 (7.1)*
BMD, z-score (SD)	0.6 (1.2)	1.0 (1.3)*	1.0 (1.3)*
Women, %	47.2	48.6	50.5
BMI (kg/m^2^), % prevalence			
<25	32.7	9.5	19.2%
25 to 30	43.2	36.0*	41.7*
>30	24.1	54.5*	39.2*
Nodal OA, % prevalence	10.3	28.8*	24.6*
Injury (knee or hip), % prevalence	16.8	32.8*	22.2*
Occupational risk factors, % prevalence			
None	61.8*	48.8	57.2
1 OA risk factor	12.5	14.3	12.1
2 OA risk factors	11.7	14.1	12.4
3+ OA risk factors	14.1	22.7*	18.3*

The number per group, mean (SD) or percentage prevalence are presented. *P <0.01, cases versus controls. OA, osteoarthritis; BMD, bone mineral density.

### Stratified analysis

Table 2 shows the results of stratified analysis of *TGFβ1 *polymorphisms, being overweight (BMI ≥25 kg/m^2^) and the risk of knee OA. For example, compared with the reference group, (that is, normal weight (BMI<25 kg/m^2^) individuals carrying the wild-type genotype (11) of SNP rs2278422, the risk for knee OA was approximately 7.0-fold higher (OR = 6.95, 95% CI 4.22, 11.47) in overweight individuals only, 1.4-fold (OR = 1.43, 95% CI 0.83, 2.43) in individuals carrying the variant allele (12/22) only and 4.7-fold higher (OR = 4.72. 95% CI 2.92, 7.64) when both risk factors were present, suggesting an antagonistic interaction between the variant allele of this polymorphism and being overweight.

**Table 2:** Risk estimates for the association of *TGFβ1 *SNPs with knee OA by being overweight (BMI >25k g/m^2^)

SNP	Gen	OW	Knee OA	Contr	Univariate			Adjusted†			Adjusted‡		
			**N**	**N**	**OR**	**(95% CI)**	***P-*value**	**OR**	**(95% CI)**	***P-*value**	**OR**	**(95% CI)**	***P-*value**

rs11466321	11	-	83	273	1.00			1.00			1.00		
	12/22	-	16	41	1.28	(0.69, 2.40)	0.436	1.19	(0.62, 2.29)	0.607	1.10	(0.54, 2.22)	0.790
	11	+	770	548	4.60	(3.52, 6.02)	<0.001	4.84	(3.66, 6.40)	<0.001	4.08	(3.02, 5.52)	<0.001
	12/22	+	170	98	5.76	(4.06, 8.18)	<0.001	5.75	(4.00, 8.28)	<0.001	4.83	(3.28, 7.11)	<0.001
													
rs8179181	11	-	11	15	1.00			1.00			1.00		
	12/22	-	87	261	0.45	(0.20, 1.03)	0.058	0.45	(0.19, 1.06)	0.068	0.53	(0.21, 1.29)	0.162
	11	+	67	49	1.86	(0.79, 4.41)	0.156	1.98	(0.79, 4.93)	0.143	2.03	(0.78, 5.25)	0.147
	12/22	+	862	617	1.90	(0.87, 4.17)	0.109	1.98	(0.86, 4.57)	0.110	2.01	(0.84, 4.78)	0.115
													
rs8105161	11	-	2	13	1.00			1.00			1.00		
	12/22	-	96	299	2.09	(0.46, 9.41)	0.338	2.27	(0.49, 10.58)	0.297	2.41	(0.50, 11.57)	0.271
	11	+	23	18	8.31	(1.66, 41.61)	0.010	9.53	(1.83, 49.78)	0.008	9.41	(1.72, 51.60)	0.010
	12/22	+	915	623	9.53	(2.14, 42.38)	0.003	10.93	(2.38, 50.21)	0.002	9.86	(2.08, 46.61)	0.004
													
rs2278422	11	-	28	113	1.00			1.00			1.00		
	12/22	-	68	197	1.39	(0.85, 2.29)	0.191	1.31	(0.78, 2.19)	0.306	1.43	(0.83, 2.48)	0.197
	11	+	351	182	7.80	(4.97, 12.25)	<0.001	7.57	(4.74, 12.08)	<0.001	6.95	(4.22, 11.47)	<0.001
	12/22	+	582	462	5.07	(3.29, 7.80)	<0.001	5.28	(3.38, 8.27)	<0.001	4.72	(2.92, 7.64)	<0.001
													
rs2241718	11	-	3	13	1.00			1.00			1.00		
	12/22	-	93	293	1.38	(0.38, 4.93)	0.625	1.37	(0.37, 5.11)	0.639	1.36	(0.35, 5.24)	0.659
	11	+	29	25	5.03	(1.28, 19.67)	0.020	5.56	(1.36, 22.75)	0.017	4.86	(1.13, 20.93)	0.034
	12/22	+	896	607	6.39	(1.81, 22.50)	0.004	6.73	(1.84, 24.66)	0.004	5.71	(1.50, 21.65)	0.010
													
rs1800468	11	-	82	258	1.00			1.00			1.00		
	12/22	-	15	54	0.87	(0.47, 1.63)	0.672	0.86	(0.45, 1.63)	0.637	0.92	(0.46, 1.84)	0.818
	11	+	795	551	4.53	(3.45, 5.94)	<0.001	4.75	(3.58, 6.31)	<0.001	4.11	(3.03, 5.56)	<0.001
	12/22	+	134	90	4.68	(3.25, 6.75)	<0.001	4.98	(3.40, 7.29)	<0.001	4.16	(2.77, 6.24)	<0.001
													
rs1800469	11	-	7	27	1.00			1.00			1.00		
	12/22	-	91	281	1.25	(0.53, 2.96)	0.614	1.33	(0.54, 3.25)	0.534	1.26	(0.50, 3.19)	0.626
	11	+	84	59	5.43	(2.22, 13.29)	<0.001	5.97	(2.36, 15.09)	<0.001	5.39	(2.05, 14.12)	0.001
	12/22	+	846	567	5.75	(2.49, 13.30)	<0.001	6.46	(2.72, 15.39)	<0.001	5.19	(2.11, 12.76)	<0.001
													
rs1982073	11	-	38	127	1.00			1.00			1.00		
	12/22	-	60	180	1.11	(0.70, 1.77)	0.649	1.13	(0.70, 1.83)	0.612	1.26	(0.75, 2.10)	0.381
	11	+	351	257	4.56	(3.07, 6.79)	<0.001	4.95	(3.28, 7.47)	<0.001	4.37	(2.82, 6.78)	<0.001
	12/22	+	569	374	5.07	(3.45, 7.45)	<0.001	5.35	(3.58, 7.97)	<0.001	4.91	(3.20, 7.53)	<0.001

†Adjusted for age and gender; ‡adjusted for age, gender, nodal OA, knee injury, occupational risk factors and bone mineral density (z-score). For OW (overweight), - indicates not overweight, + indicates overweight; *TGFβ1*, transforming growth factor β1; OA, osteoarthritis; SNP, single nucleotide polymorphism; Gen, genotye; OW, overweight; Contr, controls; OR, odds ratio.

Similar stratified analysis for hip OA is presented in Table 3. For example, for SNP rs1800468, the risk for hip OA was significantly elevated in overweight individuals only (OR = 1.71, 95% CI 1.33, 2.18) but there was no significant association with genotype 12/22 only (*P *= 0.071). However, there was a 2-fold (OR = 2.21, 95% CI 1.55, 3.15) risk for hip OA when both risk factors were present, suggesting a synergistic interaction between the variant allele of this polymorphism and being overweight.

**Table 3:** Risk estimates for the association of *TGFβ1 *SNPs with hip OA by being overweight (BMI >25 kg/m2)

SNP	Genotype	Overweight	Hip OA	Controls	Univariate			Adjusted†			Adjusted‡		
			**N**	**N**	**OR**	**(95% CI)**	***P*-value**	**ORs**	**(95% CI)**	***P*-value**	**ORs**	**(95% CI)**	***P*-value**

rs11466321	11	-	162	273	1.00			1.00			1.00		
	12/22	-	30	41	1.23	(0.74, 2.05)	0.420	1.22	(0.72, 2.06)	0.466	1.18	(0.68, 2.05)	0.552
	11	+	683	548	2.10	(1.68, 2.63)	<0.001	2.25	(1.78, 2.84)	<0.001	1.97	(1.54, 2.53)	<0.001
	12/22	+	125	98	2.17	(1.56, 3.02)	<0.001	2.22	(1.58, 3.12)	<0.001	1.92	(1.34, 2.74)	<0.001
													
rs8179181	11	-	17	15	1.00			1.00			1.00		
	12/22	-	197	261	0.67	(0.32, 1.37)	0.268	0.57	(0.27, 1.21)	0.143	0.72	(0.33, 1.56)	0.404
	11	+	43	49	0.77	(0.35, 1.73)	0.534	0.75	(0.32, 1.75)	0.505	0.79	(0.33, 1.90)	0.602
	12/22	+	728	617	1.04	(0.52, 2.11)	0.907	0.94	(0.45, 1.97)	0.871	1.03	(0.48, 2.21)	0.944
													
rs8105161	11	-	9	13	1.00			1.00			1.00		
	12/22	-	182	299	0.88	(0.37, 2.10)	0.772	0.95	(0.39, 2.31)	0.906	0.94	(0.38, 2.33)	0.890
	11	+	19	18	1.52	(0.52, 4.43)	0.438	1.78	(0.59, 5.34)	0.304	1.75	(0.57, 5.41)	0.329
	12/22	+	790	623	1.83	(0.78, 4.32)	0.165	2.09	(0.87, 5.03)	0.098	1.82	(0.74, 4.45)	0.189
													
rs2278422	11	-	66	113	1.00			1.00			1.00		
	12/22	-	126	197	1.10	(0.75, 1.60)	0.637	1.08	(0.73, 1.60)	0.703	1.07	(0.71, 1.60)	0.751
	11	+	260	182	2.46	(1.72, 3.52)	<0.001	2.55	(1.76, 3.70)	<0.001	2.26	(1.53, 3.33)	<0.001
	12/22	+	542	462	2.01	(1.45, 2.79)	<0.001	2.14	(1.52, 3.02)	<0.001	1.85	(1.29, 2.65)	0.001
													
rs2241718	11	-	9	13	1.00			1.00			1.00		
	12/22	-	182	293	0.90	(0.38, 2.14)	0.807	0.95	(0.39, 2.31)	0.905	0.95	(0.38, 2.35)	0.908
	11	+	23	25	1.33	(0.48, 3.69)	0.585	1.60	(0.56, 4.59)	0.377	1.59	(0.54, 4.67)	0.395
	12/22	+	766	607	1.83	(0.78, 4.30)	0.168	2.04	(0.85, 4.90)	0.112	1.78	(0.73, 4.36)	0.205
													
rs1800468	11	-	174	258	1.00			1.00			1.00		
	12/22	-	17	54	0.47	(0.26, 0.83)	0.010	0.52	(0.28, 0.93)	0.029	0.57	(0.31, 1.05)	0.071
	11	+	661	551	1.78	(1.43, 2.23)	<0.001	1.92	(1.52, 2.42)	<0.001	1.71	(1.33, 2.18)	<0.001
	12/22	+	140	90	2.31	(1.66, 3.20)	<0.001	2.47	(1.75, 3.47)	<0.001	2.21	(1.55, 3.15)	<0.001
													
rs1800469	11	-	13	27	1.00			1.00			1.00		
	12/22	-	178	281	1.32	(0.66, 2.62)	0.434	1.39	(0.68, 2.84)	0.362	1.37	(0.66, 2.85)	0.396
	11	+	65	59	2.29	(1.08, 4.84)	0.030	2.46	(1.13, 5.35)	0.024	2.09	(0.94, 4.65)	0.072
	12/22	+	727	567	2.67	(1.36, 5.22)	0.004	3.00	(1.50, 6.02)	0.002	2.61	(1.28, 5.35)	0.009
													
rs1982073	11	-	74	127	1.00			1.00			1.00		
	12/22	-	112	180	1.07	(0.74, 1.55)	0.729	1.11	(0.76, 1.63)	0.594	1.22	(0.82, 1.83)	0.323
	11	+	294	257	1.96	(1.41, 2.74)	<0.001	2.22	(1.57, 3.14)	<0.001	2.06	(1.44, 2.95)	<0.001
	12/22	+	498	374	2.29	(1.67, 3.14)	<0.001	2.45	(1.76, 3.40)	<0.001	2.27	(1.61, 3.20)	<0.001

For Overweight, -indicates not overweight, + indicates overweight; †adjusted for age and gender; ‡adjusted for age, gender, nodal OA, hip injury, occupational risk factors and bone mineral density (Z score); *TGFβ1*, transforming growth factor β1; SNP, single nucleotide polymorphism; OA, osteoarthritis; OR, odds ratio.

### Interaction term in the models

Table 4 shows a summary of significant results from the analysis of interaction between *TGFβ1 *polymorphisms and BMI in OA (see Additional file 1 for results on all SNPs). For knee OA, a significant antagonistic interaction on both the multiplicative and additive scale was observed for SNP rs2278422. Assuming a multiplicative scale, the OR for interaction was 0.47 (95% CI 0.26, 0.86) whilst under an additive scale, the AP was -0.61 (95% CI -0.95, -0.27), indicating that both factors acted antagonistically (RERI = -2.66, SI = 0.59) in relation to risk of knee OA, so that the AP to knee OA was 61% lower than expected from the addition of separate effects of genotype 12/22 and being overweight. Additive interaction was also observed between SNP rs11466321 and being overweight but this effect became null after adjusting for potential confounders. Furthermore, no statistically significant multiplicative interaction (ORINT = 1.08, *P *= 0.851) was found for this SNP (Table 4).

**Table 4:** Significant results from gene-environment interactions of *TGFβ1 *SNPs and BMI in OA

*TGFβ1 *SNP	OA phenotyp	Interaction	Univariate			Adjusted‡ OR		
			
			ORINT	(95% CI)	*P*-Value (LRT)	ORINT	(95% CI)	*P*-Value (LRT)
rs11466321	Knee OA	Multiplicative	0.96	(0.49, 1.91)	0.911	1.08	(0.50, 2.31)	0.851
		RERI	4.25	(2.46, 6.05)		0.67	(-0.92, 2.26)	
		AP	0.75	(0.59, 0.90)		0.14	(-0.16, 0.44)	
		Synergy Index	10.46	(1.42, 77.30)		1.21	(0.77, 1.89)	
rs2278422§	Knee OA	Multiplicative	0.47	(0.27, 0.81)	0.005	0.47	(0.26, 0.86)	0.013
		RERI	-3.09	(-5.37, -0.81)		-2.66	(-4.96, -0.36)	
		AP	-0.61	(-0.95, -0.27)		-0.56	(-0.94, -0.17)	
		Synergy Index	0.57	(0.45, 0.72)		0.59	(0.44, 0.78)	
rs1800468¶	Hip OA	Multiplicative	2.78	(1.46, 5.30)	0.001	2.27	(1.15, 4.48)	0.015
		RERI	1.06	(0.38, 1.75)		0.93	(0.19, 1.67)	
		AP	0.46	(0.24, 0.68)		0.42	(0.16, 0.68)	
		Synergy Index	5.32	(0.72, 39.11)		4.33	(0.59, 31.96)	

‡Adjusted for age, gender, nodal OA, joint injury, occupational risk factors and bone mineral density (z-score). ^§^Statistically significant with RERI <0, AP <0, and synergy index (SI) <1 indicating an antagonistic interaction. ^¶^Statistically significant with RERI >0, AP >0, and SI >1, indicating a synergetic interaction. *TGFβ1*, transforming growth factor β1; SNP, single nucleotide polymorphism; BMI, body mass index; OA, osteoarthritis; OR, odds ratio; ORINT, odds ratio for interaction; LRT, likelihood ratio test; RERI, relative excess risk due to interaction; AP, attributable proportion.

For hip OA, an additive interaction was observed between SNP rs1800468 and being overweight. Both factors acted synergistically (RERI = 0.93, SI = 4.33) to increase the risk of hip OA, so that among overweight persons carrying the variant allele, 42% (AP = 0.42; 95% CI 0.16, 0.68) of hip OA risk was attributable to the action of both exposures as compared with the contribution of each of the two risk factors added to each other. A significant multiplicative interaction (ORINT = 2.27, *P *= 0.015) for this SNP was also found (Table 4).

## Discussion

This is the first study to examine possible interactions between increased BMI and *TGFβ1 *gene polymorphisms for knee and hip OA. We found significant additive and multiplicative interaction between being overweight and the variant allele of *TGFβ1 *SNP rs2278422 in knee OA but in hip OA these interactions were indicated by *TGFβ1 *SNP rs1800468.

Obesity is a known risk for OA and it has been postulated that in addition to the impact on joint mechanics [22,23] there may be a link between dysfunctional metabolism and joint damage via adipose-secreted cytokines termed adipokines, which are known regulators of metabolic homeostasis [24,25]. *In vitro *experiments with human and animal culture cells as well as *in vivo *animal studies have shown an increased expression of *TGFβ1 *in response to mechanical stimuli in a number of cell and tissue types [6,7,12,13]. Furthermore, experimental models have found excess endogenous TGFβ in association with both chondrocyte synthesis and osteophyte formation [6,7]. Leptin may also play a key role in the development of OA mainly through mechanisms that modulate TGFβ function in maintaining cartilage and bone integrity [24,25]. There is evidence of increased leptin expressions in synovial fluid and in cartilage and osteophytes of patients with OA [24]. Therefore, it is plausible that excessive biomechanical loading of joints or stimulation by adipokines such as leptin may stimulate the up-regulation of endogenous *TGFβ1*, leading to formation of new fibrocartilage that then undergoes endochondral ossification to become osteophytes. This hypothesis may partly explain the antagonistic interaction that was observed in this study between being overweight and the variant allele of the *TGFβ1 *polymorphism rs2278422 in knee OA (Table 4). A previously reported association using the GOAL population found the heterozygous genotype of the *TGFβ1 *polymorphism rs2278422 to be associated with reduced risk of knee OA [9].

On the other hand, we found increased BMI to interact with the variant allele of SNP rs1800468 to increase the risk of hip OA (Table 4). This finding suggests that a greater body weight increased the risk of hip OA in this population, but overweight individuals with the variant allele of *TGFβ1 *SNP rs1800468 appeared to have an even greater risk (Table 3). Our previous association analysis did not find a statistically significant association between SNP rs1800468 and hip OA, further demonstrating that on its own, it did not influence risk for hip OA in this population [9]. These different findings at the knee and hip further support consideration of OA at these two sites to be discrete subsets in terms of risk factor profile.

The functional relevance of these *TGFβ1 *variants remains unknown. The location of SNP rs1800468 in the promoter genomic region (5'UTR) suggests that it may play a role in gene regulation and signal sequence. SNP rs2278422 resides in intron 8, a region of unknown biological importance. Nevertheless, we cannot exclude the possibility that the observed interactions may be due to linkage disequilibrium (LD). For example, SNP rs1800468 has been found to be in LD with SNP rs1800469 [26] and SNP rs1800469 is also reported to be in strong LD with rs1982073 [27]. Therefore, it is conceivable that these interactions may result from this SNP being tightly linked to another susceptibility genetic variant of biological importance.

Some of the strengths of this study include: a well-characterised cohort; a sufficient sample size to detect modest interactions in a case-control design; and radiographic data on the control population. In addition, confounding resulting from population stratification was minimised, as all participants were Caucasian and were recruited from a homogenous geographical area.

However, this study also has several caveats. First, we recruited hospital cases and controls which may have led to selection bias. Cases were patients with clinically severe symptoms of OA sufficient to warrant hospital referral, so these findings may not be extrapolated to the full spectrum of OA patients. Second, controls were recruited from IVU lists, and hence are more likely to suffer from kidney disease, including malignancy. Although none of these conditions have any known positive or negative association with OA, controls with these diagnoses may be positively or negatively associated with the *TGFβ1 *gene and result in exaggerated risks. Nevertheless, this was unlikely to have affected our findings since the *TGFβ1 *polymorphisms we examined did not deviate from Hardy-Weinberg Equilibrium (HWE) in the control population. Third, the study measured cross-sectional weight and height at the end-stage of the disease. Therefore, it is not possible to determine whether being overweight preceded OA or was the consequence of it. Despite this limitation, BMI provided a more reliable proxy indicator for mechanical stresses compared to physical activity and occupational risk factors, which were more prone to recall bias. Fourth, we carried out a number of multiple statistical tests but no adjustments were made, which may have resulted in false positive significant interactions. A common approach used in many studies to attempt to reduce the number of polymorphisms tested is to restrict gene-environment interaction analysis only to gene variants that show significant association with the outcome. However, this approach gives little consideration to a large environmental risk component such as BMI in OA. Instead, we have described and provided the tests of significance used as recommended by Pernerger [28] as a way of dealing with multiple testing. Finally, our results were not replicated in a separate independent dataset, therefore, future studies are needed to validate these findings in different populations and study settings.

## Conclusions

In conclusion, we have identified for the first time potential interaction between *TGFβ1 *and being overweight in large joint OA. The results were supported by both multiplicative and additive models and appeared to differ between knee and hip OA. The results require replication in other populations and if confirmed, further molecular studies are warranted to better understand the underlying mechanisms responsible for such interactions in OA.

## Abbreviations

AP: attributable proportion; BMD: bone mineral density; BMI: body mass index; GOAL: study Genetics of OA and Lifestyle study; HWE; Hardy-Weinberg equilibrium; IVU: intravenous urography; KL: Kellgren-Lawrence; LD: linkage disequilibrium; LRT: likelihood ratio test; OA: osteoarthritis; OR: odds ratio; ORINT: odds ratio for interaction; RERI: relative excess risk due to interaction; SI: synergy index; SNP: single nucleotide polymorphism; *TGFβ1*: *transforming growth factor beta 1*; TJR: total joint replacement; UTR: untranslated region.

## Competing interests

Rose A Maciewicz owns stock or stock options in AstraZeneca and has submitted patent finding on the OA gene. All other authors declare no competing interests.

## Authors' contributions

SGM participated in the design of the study, collected, analyzed and interpreted the data, and drafted the manuscript. SD participated in the design of the study, acquisition of data, and critically edited and revised the manuscript. WZ participated in the design of the study and analysis and interpretation of the data, and critically edited and revised the manuscript. RAM conceived and designed the study, interpreted the data, and critically edited and revised the manuscript. KM and MD conceived and designed the study, participated in the analysis and interpretation of the data, and critically edited and revised the manuscript. All authors read and approved the final manuscript for publication.

## Supplementary Material

Additional file 1 **Additive and multiplicative gene-environment interactions in knee and hip osteoarthritis**. This file contains two tables with results on additive and multiplicative gene-environment interactions among transforming growth factor (TGF)β1 single nucleotide polymorphisms (SNPs), body mass index (BMI) and osteoarthritis (OA). Table S1: Gene-environment interactions among *TGFβ1 *SNPs, BMI and knee OA. Table S2: Gene-environment interactions among *TGFβ1 *SNPs, BMI and hip OA. Click here for file
