# Supplementary material for: Gene-environment interaction between body mass index and transforming growth factor beta 1 (TGFβ1) gene in knee and hip osteoarthritis
Source: Arthritis Res Ther. 2013 Apr 18;15(2):R52. doi: 10.1186/ar4214 (PMC4060375; doi:10.1186/ar4214)
Supplement: Additional file 1 — Additive and multiplicative gene-environment interactions in knee and hip osteoarthritis. This file contains two tables with results on additive and multiplicative gene-environment interactions among transforming growth factor (TGF)β1 single nucleotide polymorphisms (SNPs), body mass index (BMI) and osteoarthritis (OA). Table S1: Gene-environment interactions among TGFβ1 SNPs, BMI and knee OA. Table S2: Gene-environment interactions among TGFβ1 SNPs, BMI and hip OA. [file ar4214-S1.DOC]

**Additional file 1: Additive and multiplicative gene-environment interactions in knee and hip osteoarthritis**

**Table S1: Gene-environment interactions among TGFβ1 SNPs, BMI and knee** OA

| **TGFβ1 SNP** | **Interaction** | **Univariate** | | | |  | **Adjusted‡ ORs** | | | |
| --- | --- | --- | --- | --- | --- | --- | --- | --- | --- | --- |
|  |  | **ORINT** | **ORINT 95% C.I.** | | **P Value (LRT)** |  | **ORINT** | **ORINT 95% C.I.** | | **P Value (LRT)** |
|  |  |  |  |  |  |  |  |  |  |  |
| rs11466321 | Multiplicative | 0.96 | (0.49, | 1.91) | 0.911 |  | 1.08 | (0.50, | 2.31) | 0.851 |
|  | RERI | **4.25** | **(2.46,** | **6.05)** |  |  | 0.67 | (-0.92, | 2.26) |  |
|  | AP | **0.75** | **(0.59,** | **0.90)** |  |  | 0.14 | (-0.16, | 0.44) |  |
|  | SI | **10.46** | **(1.42,** | **77.30)** |  |  | 1.21 | (0.77, | 1.89) |  |
|  |  |  |  |  |  |  |  |  |  |  |
| rs8179181 | Multiplicative | 2.25 | (0.91, | 5.53) | 0.083 |  | 1.88 | (0.70, | 5.09) | 0.217 |
|  | RERI | 0.59 | (-0.19, | 1.37) |  |  | 0.45 | (-0.54, | 1.45) |  |
|  | AP | 0.31 | (-0.24, | 0.86) |  |  | 0.23 | (-0.38, | 0.83) |  |
|  | SI | 2.84 | (0.03, | 274.46) |  |  | 1.82 | (0.12, | 27.12) |  |
|  |  |  |  |  |  |  |  |  |  |  |
| rs8105161 | Multiplicative | 0.55 | (0.11, | 2.81) | 0.452 |  | 0.43 | (0.08, | 2.43) | 0.317 |
|  | RERI | 0.15 | (-5.25, | 5.55) |  |  | -0.57 | (-7.40, | 6.26) |  |
|  | AP | 0.02 | (-0.56, | 0.59) |  |  | -0.06 | (-0.73, | 0.61) |  |
|  | SI | 1.02 | (0.53, | 1.95) |  |  | 0.94 | (0.47, | 1.89) |  |
|  |  |  |  |  |  |  |  |  |  |  |
| **rs2278422** | Multiplicative | **0.47** | **(0.27,** | **0.81)** | **0.005** |  | **0.47** | **(0.26,** | **0.86)** | **0.013** |
|  | RERI | **-3.09** | **(-5.37,** | **-0.81)** |  |  | **-2.66** | **(-4.96,** | **-0.36)** |  |
|  | AP | **-0.61** | **(-0.95,** | **-0.27)** |  |  | **-0.56** | **(-0.94,** | **-0.17)** |  |
|  | SI | **0.57** | **(0.45,** | **0.72)** |  |  | **0.59** | **(0.44,** | **0.78)** |  |
|  |  |  |  |  |  |  |  |  |  |  |
| rs2241718 | Multiplicative | 0.93 | (0.23, | 3.71) | 0.912 |  | 0.87 | (0.20, | 3.82) | 0.848 |
|  | RERI | 0.99 | (-1.81, | 3.80) |  |  | 0.65 | (-2.25, | 3.56) |  |
|  | AP | 0.16 | (-0.32, | 0.63) |  |  | 0.12 | (-0.44, | 0.67) |  |
|  | SI | 1.23 | (0.60, | 2.51) |  |  | 1.16 | (0.52, | 2.62) |  |
|  |  |  |  |  |  |  |  |  |  |  |
| rs1800468 | Multiplicative | 1.18 | (0.59, | 2.35) | 0.633 |  | 1.10 | (0.51, | 2.35) | 0.811 |
|  | RERI | 0.27 | (-1.19, | 1.73) |  |  | 0.17 | (-1.31, | 1.64) |  |
|  | AP | 0.06 | (-0.24, | 0.36) |  |  | 0.04 | (-0.31, | 0.39) |  |
|  | SI | 1.08 | (0.72, | 1.62) |  |  | 1.06 | (0.66, | 1.70) |  |
|  |  |  |  |  |  |  |  |  |  |  |
| rs1800469 | Multiplicative | 0.84 | (0.33, | 2.13) | 0.709 |  | 0.76 | (0.28, | 2.09) | 0.596 |
|  | RERI | 0.02 | (-2.10, | 2.14) |  |  | -0.44 | (-2.86, | 1.99) |  |
|  | AP | 0.00 | (-0.37, | 0.37) |  |  | -0.09 | (-0.53, | 0.36) |  |
|  | SI | 1.00 | (0.64, | 1.57) |  |  | 0.90 | (0.55, | 1.48) |  |
|  |  |  |  |  |  |  |  |  |  |  |
| rs1982073 | Multiplicative | 1.00 | (0.60, | 1.66) | 1.000 |  | 0.89 | (0.51, | 1.57) | 0.696 |
|  | RERI | 0.41 | (-0.69, | 1.51) |  |  | 0.26 | (-0.89, | 1.41) |  |
|  | AP | 0.08 | (-0.14, | 0.30) |  |  | 0.05 | (-0.18, | 0.29) |  |
|  | SI | 1.11 | (0.82, | 1.50) |  |  | 1.07 | (0.78, | 1.47) |  |

ORINT, odds ratio for interaction; LRT , likelihood ratio test

RERI, relative excess risk due to interaction; AP, attributable proportion due to interaction; Synergy Index (SI)

‡ adjusted for age, gender, nodal OA, joint injury, occupation risk factors and BMD (Z score)

§ Statistically significant with RERI<0 AP<0, and SI<1 indicating an antagonistic interaction

¶ Statistically significant with RERI>0 AP>0, and SI>1 indicating a synergetic interaction

**Table S2: Gene-environment interactions among TGFβ1 SNPs, BMI and hip OA**

| **TGFβ1 SNP** | **Interaction** | **Univariate** | | | |  | **Adjusted‡ ORs** | | | |
| --- | --- | --- | --- | --- | --- | --- | --- | --- | --- | --- |
|  |  | **ORINT** | **ORINT 95% C.I.** | | **P Value (LRT)** |  | **ORINT** | **ORINT 95% C.I.** | | **P Value (LRT)** |
|  |  |  |  |  |  |  |  |  |  |  |
| rs11466321 | Multiplicative | 0.83 | (0.46, | 1.49) | 0.533 |  | 0.82 | (0.44, | 1.54) | 0.545 |
|  | RERI | -0.18 | (-1.06, | 0.69) |  |  | -0.24 | (-1.13, | 0.64) |  |
|  | AP | -0.09 | (-0.50, | 0.33) |  |  | -0.13 | (-0.61, | 0.35) |  |
|  | SI | 0.86 | (0.43, | 1.72) |  |  | 0.79 | (0.34, | 1.82) |  |
|  |  |  |  |  |  |  |  |  |  |  |
| rs8179181 | Multiplicative | 2.02 | (0.88, | 4.65) | 0.099 |  | 1.81 | (0.73, | 4.45) | 0.198 |
|  | RERI | 0.60 | (0.15, | 1.05) |  |  | 0.52 | (-0.02, | 1.05) |  |
|  | AP | 0.58 | (-0.17, | 1.32) |  |  | 0.50 | (-0.32, | 1.32) |  |
|  | SI | -0.07 | n/a | |  |  | -0.06 | n/a | |  |
|  |  |  |  |  |  |  |  |  |  |  |
| rs8105161 | Multiplicative | 1.37 | (0.46, | 4.05) | 0.575 |  | 1.11 | (0.35, | 3.50) | 0.862 |
|  | RERI | 0.43 | (-0.70, | 1.55) |  |  | 0.15 | (-1.31, | 1.60) |  |
|  | AP | 0.23 | (-0.49, | 0.95) |  |  | 0.08 | (-0.75, | 0.91) |  |
|  | SI | 2.06 | 0.04, | 116.27) |  |  | 1.21 | (0.11, | 12.89) |  |
|  |  |  |  |  |  |  |  |  |  |  |
| rs2278422 | Multiplicative | 0.75 | (0.48, | 1.16) | 0.199 |  | 0.77 | (0.48, | 1.23) | 0.271 |
|  | RERI | -0.53 | (-1.27, | 0.21) |  |  | -0.48 | (-1.25, | 0.28) |  |
|  | AP | -0.27 | (-0.60, | 0.07) |  |  | -0.26 | (-0.64, | 0.12) |  |
|  | SI | 0.65 | (0.43, | 1.00) |  |  | 0.64 | (0.38, | 1.07) |  |
|  |  |  |  |  |  |  |  |  |  |  |
| rs2241718 | Multiplicative | 1.53 | (0.54, | 4.34) | 0.428 |  | 1.18 | (0.39, | 3.54) | 0.769 |
|  | RERI | 0.60 | (-0.27, | 1.46) |  |  | 0.25 | (-0.96, | 1.46) |  |
|  | AP | 0.33 | (-0.31, | 0.96) |  |  | 0.14 | (-0.61, | 0.89) |  |
|  | SI | 3.64 | (0, | 5,574.24) |  |  | 1.46 | (0.09, | 24.65) |  |
|  |  |  |  |  |  |  |  |  |  |  |
| **rs1800468** | Multiplicative | **2.78** | (**1.46,** | **5.30)** | **0.001** |  | **2.27** | (**1.15,** | **4.48**) | **0.015** |
|  | RERI | 1.06 | (0.38, | 1.75) |  |  | 0.93 | (0.19, | 1.67) |  |
|  | **AP** | **0.46** | (**0.24,** | **0.68**) |  |  | **0.42** | (**0.16,** | **0.68**) |  |
|  | SI | 5.32 | (0.72, | 39.11) |  |  | 4.33 | (0.59, | 31.96) |  |
|  |  |  |  |  |  |  |  |  |  |  |
| rs1800469 | Multiplicative | 0.88 | (0.41, | 1.93) | 0.757 |  | 0.91 | (0.40, | 2.09) | 0.826 |
|  | RERI | 0.06 | (-1.03, | 1.14) |  |  | 0.14 | (-0.94, | 1.22) |  |
|  | AP | 0.02 | (-0.39, | 0.44) |  |  | 0.05 | (-0.38, | 0.49) |  |
|  | SI | 1.04 | (0.52, | 2.08) |  |  | 1.10 | (0.49, | 2.45) |  |
|  |  |  |  |  |  |  |  |  |  |  |
| rs1982073 | Multiplicative | 1.09 | (0.71, | 1.67) | 0.694 |  | 0.90 | (0.57, | 1.43) | 0.653 |
|  | RERI | 0.25 | (-0.32, | 0.83) |  |  | -0.01 | (-0.69, | 0.67) |  |
|  | AP | 0.11 | (-0.15, | 0.37) |  |  | -0.01 | (-0.31, | 0.29) |  |
|  | SI | 1.24 | (0.69, | 2.23) |  |  | 0.99 | (0.58, | 1.68) |  |

ORINT, odds ratio for interaction; LRT, likelihood ratio test

RERI, relative excess risk due to interaction; AP, attributable proportion due to interaction; Synergy Index (SI)

‡ adjusted for age, gender, nodal OA, joint injury, occupation risk factors and BMD (Z score)

§ Statistically significant with RERI<0 AP<0, and SI<1 indicating an antagonistic interaction

¶ Statistically significant with RERI>0 AP>0, and SI>1 indicating a synergetic interaction
